# Alterations of mRNA and lncRNA profiles associated with the extracellular matrix and spermatogenesis in goats

**DOI:** 10.5713/ab.21.0259

**Published:** 2021-08-25

**Authors:** Haolin Chen, Xiaomeng Miao, Jinge Xu, Ling Pu, Liang Li, Yong Han, Fengxian Mao, Youji Ma

**Affiliations:** 1 College of Animal Science and Technology, Gansu Agricultural University, Lanzhou, 730000, China; 2 Institute of Animal Husbandry and Veterinary, Guizhou Academy of Agricultural Sciences, Guizhou, 550000, China; 3 Guizhou Province Livestock and Poultry Genetic Resources Management Station, Guiyang, Guizhou, 550000, China

**Keywords:** Goat, LncRNA, mRNA, Puberty, Testis

## Abstract

**Objective:**

Spermatozoa are produced within the seminiferous tubules after sexual maturity. The expression levels of mRNAs and lncRNAs in testicular tissues are different at each stage of testicular development and are closely related to formation of the extracellular matrix (ECM) and spermatogenesis. Therefore, we set out to study the expression of lncRNAs and mRNAs during the different developmental stages of the goat testis.

**Methods:**

We constructed 12 RNA libraries using testicular tissues from goats aged 3, 6, and 12 months, and studied the functions of mRNAs and lncRNAs using the gene ontogeny (GO) and Kyoto encyclopedia of genes and genomes (KEGG) databases. Relationships between differentially expressed genes (DEGs) were analyzed by lncRNA-mRNA co-expression network and protein-protein interaction network (PPI). Finally, the protein expression levels of matrix metalloproteinase 2 (MMP2), insulin-like growth factor 2 (IGF2), and insulin-like growth factor-binding protein 6 (IGFBP6) were detected by western blotting.

**Results:**

We found 23, 8, and 135 differentially expressed lncRNAs and 161, 12, and 665 differentially expressed mRNAs that were identified between 3 vs 6, 6 vs 12, and 3 vs 12 months, respectively. GO, KEGG, and PPI analyses showed that the differential genes were mainly related to the ECM. Moreover, *MMP2* was a hub gene and co-expressed with the lncRNA *TCONS-0002139* and *TCONS-00093342*. The results of quantitative reverse-transcription polymerase chain reaction verification were consistent with those of RNA-seq sequencing. The expression trends of MMP2, IGF2, and IGFBP6 protein were the same as that of mRNA, which all decreased with age. IGF2 and MMP2 were significantly different in the 3 vs 6-month-old group (p<0.05).

**Conclusion:**

These results improve our understanding of the molecular mechanisms involved in sexual maturation of the goat testis.

## INTRODUCTION

Mammalian testicular development and spermatogenesis are complex processes regulated by the transcriptome; they are dynamic and staged [1]. Testis-specific genes play a crucial role in male reproduction by influencing spermatogenesis and fertility [2]. Optimum fertility levels are critical to individual enterprises and to the livestock industry as a whole [3]. Past breeding efforts demonstrate that genetic selection is a highly successful tool for improving livestock populations [4]. Therefore, a comprehensive understanding of the molecular mechanisms at play within testicular tissues during goat sexual maturation will significantly promote the future success of goat breeding.

As an important part of the seminiferous tubule wall, the extracellular matrix (ECM) directly affects spermatogenesis [5]. Matrix metalloproteinases (MMPs) are a group of endopeptidases that function in the degradation of collagen in the ECM and during the regulation of homeostasis in the ECM [6]. They play an essential role in spermatogenesis [7] and remodeling by interacting with insulin-like growth factor-binding proteins (IGFBPs) and insulin-like growth factor (IGF) [8]. The matrix metalloproteinase 2 (MMP2) is an extracellular zinc protease that plays a vital role in testicular maturation and spermatogenesis [9]. In addition, IGF2 and IGFBP6 can bind with the ECM to affect a variety of cell functions [10].

Long non-coding RNAs (lncRNAs) are highly expressed non-coding RNAs that play an essential role in regulating gene expression in organisms [11], including during spermatogenesis [12]. For instance, in the process of spermatocyte division and differentiation into mature sperm, many lncRNAs are involved in synchronising the expression of specific genes to achieve regulatory purposes [13]. lncRNAs have been studied in the testicular tissues of livestock and poultry, including chicken [14], sheep [15], and pigs [16]. However, the comparative analysis of lncRNAs and mRNAs in prepubertal, pubertal, and post-pubertal testicular tissues of goats is unclear.

We used RNA-seq to evaluate the differential expression profiles of lncRNAs and mRNAs in goat testicular tissues to explore the molecular mechanisms of ECM formation and spermatogenesis. Our purpose was to both enrich our understanding of the goat genome and also to provide a basis for further research on the function of candidate genes. We used RNA-seq to research lncRNA and mRNA expression levels in the testicular tissue of goats aged 3, 6, and 12 months; these ages represent the three growth stages of prepubertal, pubertal, and post-pubertal, respectively. In addition, we further studied the protein expressions of MMP2, IGF2, and IGFBP6 by western blotting. Our research may provide new insights into the molecular mechanisms of testicular development in the goat and provide a basis for further exploration of goat breeding, spermatogenesis, and marker screening, among other roles.

## MATERIALS AND METHODS

### Ethics statement

All experiments involving goats were conducted in strict compliance with the relevant guidelines set by the Ethics Committee of Guizhou Animal Husbandry and Veterinary Research Institute (JXYJS-20190312). All tests were conducted in accordance with the relevant guidelines and regulations formulated by the Ministry of Agriculture, People’s Republic of China.

### Sample collection and RNA isolation

The 3, 6, and 12-month-old male Guizhou Black Goats were obtained from a goat farm in Weining County (Guizhou, China). All experimental animals were euthanized; the anesthesia program gave 2.5% thiopental sodium general anesthesia at a dose of 10 mg/kg, followed by electrocution. Four healthy goats were selected for each age stage. Tissue samples for the extraction of RNA and protein were removed from testicular tissue and immediately frozen in liquid nitrogen, transported to the laboratory, and stored in the refrigerator at −80°C.

Each frozen tissue sample was ground into a fine powder in liquid nitrogen, and the total RNA was extracted with Trizol reagent according to the manufacturer’s instructions. Then the concentration, purity, and integrity of total RNA were detected. RNA concentration was measured using a Qubit RNA Assay Kit in a Qubit 2.0 Flurometer (Life Technologies, Carlsbad, CA, USA). RNA purity was checked using a NanoPhotometer spectrophotometer (IMPLEN, Palo Alto, CA, USA). RNA integrity was assessed using the RNA Nano 6000 Assay Kit of the bioanalyzer 2100 system (Agilent Technologies, Palo Alto, CA, USA).

### Library preparation and RNA sequencing

After qualifying, rRNA removal, library preparation, and examination of library quality was performed. After passing the quality inspection, computer sequencing was undertaken. The raw reads obtained by sequencing were sequentially removed from the reads with adapter; thereafter, clean reads were obtained after those reads with a proportion of undefinable base information more significant than 10% and low-quality reads.

### Identification and prediction of differentially expressed genes

The reference genome index (https://www.ncbi.nlm.nih.gov/genome/?term=Capra+aegagrus+hircus) was built using Bowtie (v2.0.6) [17], and paired-end clean reads were aligned to the reference genome using TopHat (v2.0.9) [18]. Basic screening mainly included the selection of transcripts with length ≥200 bp and exon number ≥2; the reads of each transcript with minimum coverage ≥3 were calculated by StringTie; similar or identical transcripts were screened by comparing them with known non-lncRNAs and non-mRNAs using Cuffcompare in goats; by comparing with known mRNAs and using class_code information in Cuffcompare results, candidate transcripts of different types were screened (lincRNA, intronic lncRNA, anti-sense lncRNA, and sense-overlapping lncRNA). We applied four approaches to the analysis of Coding potential: the Coding-Non-Coding-Index (CNCI) [19]; the Coding Potential Calculator (CPC) [20]; Pfam [21], and the Coding Potential Assessment Tool (CPAT) [22].

### Target gene prediction and enrichment analysis

The target genes of lncRNAs were predicted by cis- or trans-action. Cis-action screened out the protein coding genes adjacent to lncRNA (upstream and downstream 100 K) as its target genes. Trans-action target genes were predicted by selecting the Pearson correlation coefficient (PCC) of lncRNAs and protein-coding genes between samples >0.6, and comparing the base complementary coordination relationship between lncRNAs and mRNAs.

The FPKMs (Reads Per Kilobase of exon model per Million mapped reads) of lncRNA and coding genes in each sample were calculated using Cuffdiff (v2.1.1) [23]. The FPKM value was used to estimate the expression levels of genes. Gene ontogeny (GO) enrichment analysis of differentially expressed genes (DEG) was conducted using the goseq R package [24]. Kyoto encyclopedia of genes and genomes (KEGG) pathways were detected using KOBAS (KEGG Orthology Based Annotation System) [25]. Corrected p-values <0.05 were considered significantly enriched by DEGs.

### PPI network and lncRNA–mRNA co-expression network analysis

The protein–protein interaction (PPI) network based on DEGs was significantly enriched in GO terms between 3 vs 6 months of age. The PPI network was obtained through an online analysis tool (https://www.string-db.org/). By calculating the expression correlation between lncRNAs and genes encoded by custom scripts, and using WGCNA to cluster genes from different samples, standard expression modules were found [26]. Subsequently, their functions were analyzed using functional enrichment analysis. The co-expression of lncRNAs-mRNAs was analyzed by calculating PCC between the encoding gene and the specific expression level of lncRNAs. The differentially expressed lncRNAs were correlated with the predicted cis- or trans-acting target mRNAs. The visualization of gene interaction was realized using Cytoscape software (v3.3.0) (The Cytoscape Consortium, USA).

### Quantitative reverse-transcription polymerase chain reaction validation

We identified 11 differentially expressed mRNAs and five differentially expressed lncRNAs. Primers were designed and detected using the NCBI Pick Primers and Primer-BLAST tools, respectively. Primer information is listed in Table 1. The 2^−ΔΔCt^ quantitative method was used to calculate the relative expression level of mRNAs and lncRNAs [27], and normalization took place using the housekeeping gene glyceraldehyde-3-phosphate dehydrogenase (GAPDH). The total RNA of goat testicular tissue was isolated by TRIzol (Invitrogen Life Technologies, Carlsbad, CA, USA). cDNAs were synthesized from RNA using a PrimeScript RT Kit with gDNA Eraser (Takara, Dalian, China). Quantitative polymerase chain reaction (qPCR) was performed with SYBR Green Master mix (Roche Applied Science, Mannheim, Germany). The 20 μL reaction solution contained 9 μL SYBR, 2 μL cDNA, 1 μL each of the forward and reverse primers, and 7 μL ddH_2_O. The reaction system consisted of a holding stage of 5 min at 50°C, and 10 min at 95°C, 40 cycles of 95°C for 15 s, 60°C for 30 s, and 72°C for 30 s; and a final stage at 95°C for 15 s, 60°C for 30 s, and 95°C for 15 s.

### Western blotting analyses

The testicular tissue samples were washed three times with phosphate buffered saline (Servicebio, China), then cut into small pieces and place in an equalizer with 12,000 g of homogenate for 10 min. The supernatant was collected, and the protein concentration was measured using a BCA protein concentration assay kit according to the manufacturer’s instructions (Servicebio, China) and normalized to GAPDH levels. Transfer condition: 200 mA transfer for 1 h. Primary antibodies against IGF2, IGFBP6, and MMP2 (diluted 1:500, 1:1,000 and 1:1,000; cat. no: ab170304, ab109765, and ab97779, respectively [Abcam, Cambridge, UK]), and GAPDH (diluted 1:30,000; cat. no: GB12002, Proteintech Group, Inc., Wuhan, China) were incubated at 4°C for 3 h. Subsequently, the goat anti-rabbit horseradish peroxidase immunoglobulin G secondary antibody (diluted 1:5,000; Servicebio, cat. no: GB23303, China) was incubated at room temperature for 30 min. Images were acquired using a Perform Electrochemiluminescence kit (Servicebio, China) following the manufacturer’s instructions. For image analysis, films were scanned, organized, and desaturated using Adobe Photoshop (Adobe, Santa Clara, CA, USA). The optical density of the target band was analyzed using the Alpha processing system (Alpha Innotech, Shanghai, China).

### Statistical analysis

The original data was organized in Microsoft Excel 2010. The one-way analysis of variance (age factor) analytical method was performed to compare means using SPSS 21.0 software (SPSS, Chicago, USA). All results were expressed as mean±standard deviation. Similar letters indicate no significant difference (p>0.05), while different letters indicate a significant difference (p<0.05).

## RESULTS

### Testis transcriptome characterization and lncRNA identification

**Figure 1:**
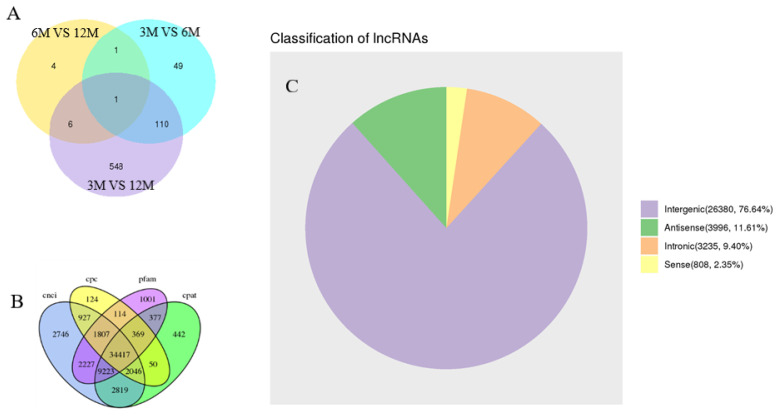
Venn diagram and pie chart of differentially expressed genes. (A) Pie chart of differentially expressed mRNAs. (B) Venn diagram according to four tools used for coding protein analysis. (C) Statistical analysis of different lncRNAs. 3M, 3 months old; 6M, 6 months old; 12M, 12 months old.

We constructed 12 cDNA libraries using total testicular RNA from four individuals from each of the three specific stages of growth and development in goats. Each sample contained approximately 7.52 Gb of high-quality sequence data. The data has been deposited into the NCBI database (NCBI Accession No: PRJNA613301). Pairwise comparison analysis of different groups showed that 161, 12, and 665 differentially expressed mRNAs were present in the testicular tissues of goats between 3 vs 6, 6 vs 12, and 3 vs 12 months, respectively. In addition, there was a common differential gene, *MMP2*, among the three groups of differential genes. In total, we intersected the results of CPC/CNCI/PFAM/CPAT and obtained lncRNAs for subsequent analysis. Results including 26,380 lincRNAs (76.64%), 3,996 anti-sense lncRNAs (11.61%), 3,235 intronic lncRNAs (9.40%), and 808 sense lncRNAs (2.35%) (Figure 1).

### Differentially expressed gene analysis

**Figure 2:**
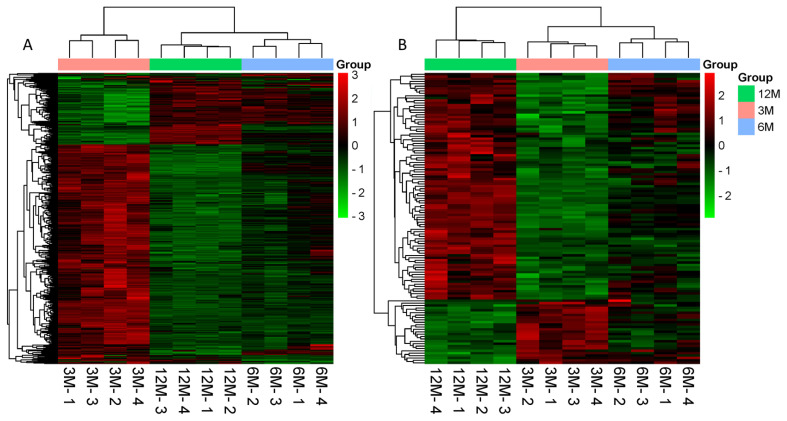
Hierarchical heat map of a differential expression cluster graph. (A) Clustering heat map of differentially expressed mRNAs. (B) Clustering heat map of differentially expressed lncRNAs. Red indicates highly expressed genes and green indicates lowly expressed genes. 3M, 3 months old; 6M, 6 months old; 12M, 12 months old.

A total of 161 (105 up, 56 down), 12 (9 up, 3 down), and 665 (512 up, 153 down) differentially expressed mRNAs were identified. Furthermore, 23 (5 up, 18 down), 8 (5 up, 3 down), and 135 (33 up, 102 down) differentially expressed lncRNAs were present in 3 vs 6, 6 vs 12, and 3 vs 12-month-old goat testes, respectively. The differentially expressed mRNAs and lncRNAs were clustered together owing to their similar expression profiles, which are shown in Figure 2A and Figure 2B as heat maps, respectively.

### Functional enrichment analysis

**Figure 3:**
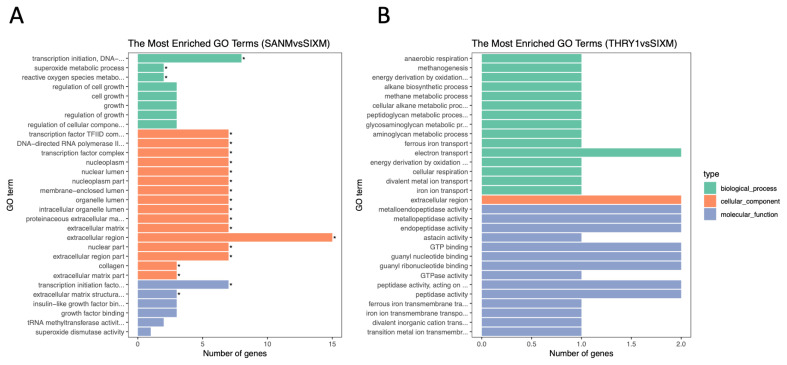
The top 30 GO terms listed in the GO analysis of differentially expressed mRNAs. (A) 3 vs 6 months of age. (B) 6 vs 12 months of age. * Indicates significant enrichment (p<0.05). GO, gene ontology.

According to GO analysis results of DEG, a total of 21 GO terms were significantly enriched between 3 vs 6 months of age (Figure 3A). In comparison, there was no significant enrichment in GO terms between 6 vs 12 months of age (Figure 3B). Based on GO analysis of biological processes, we found that these DEGs were significantly enriched in three GO terms (reactive oxygen species metabolic process, superoxide metabolic process, and transcription initiation DNA-dependent). In addition, according to the GO analysis result of molecular function, the transcription initiation factor activity and ECM structural constituent were also significantly enriched. Moreover, the level of significant enrichment in cell composition was the largest, with 16 GO terms, including transcription factor complex, DNA-directed RNA polymerase II, membrane-enclosed lumen, organelle lumen, intracellular organelle lumen, ECM, proteinaceous ECM, and extracellular region (Figure 3A).

**Figure 4:**
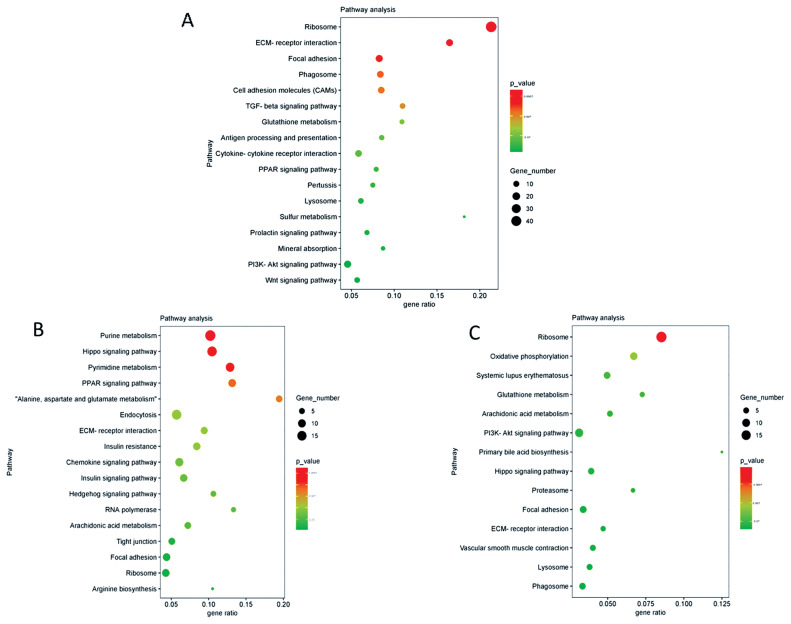
KEGG analysis of 3 vs 12 month old differentially expressed genes and lncRNA target genes. (A) Differentially expressed genes. (B) Differentially expressed lncRNA cis target genes. (C) Differentially expressed lncRNA trans target genes; KEGG, Kyoto encyclopedia of genes and genomes.

The significantly enriched KEGG pathway analysis of mRNA revealed that many pathways were related to spermatogenesis; as an example, the ECM-receptor interaction signaling pathway was present. KEGG pathway analysis of lncRNA cis- and trans-target genes revealed that many significant enrichment pathways were consistent with the results of mRNA enrichment (Figure 4); including ECM-receptor interaction signaling pathway, focal adhesion, and ribosome.

### PPI network and Co-expression network construction

**Figure 5:**
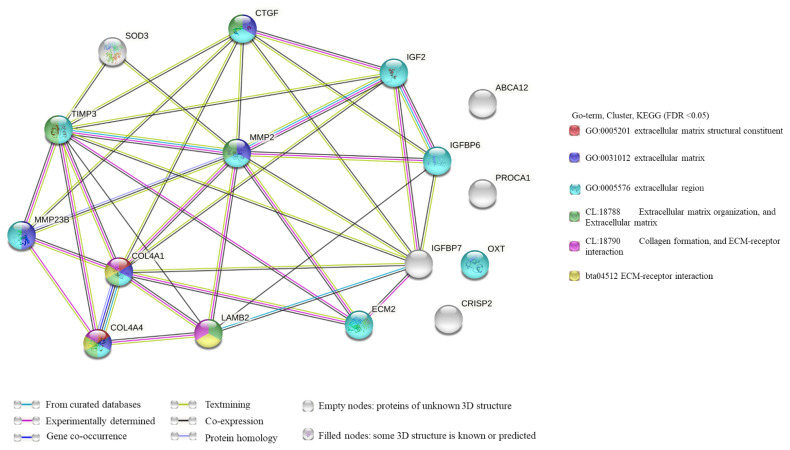
PPI network analysis of differentially expressed genes significantly enriched in GO terms. Line color represents different types of interaction evidence. PPI, protein–protein interaction; GO, gene ontology.

PPI network analysis of DEGs from significantly enriched GO terms. There were 38 edges and 16 nodes in the network (Figure 5). According to the results of the PPI network analysis, *MMP2* was the hub gene (degree = 29). In addition, among these DEGs, most were related to the ECM (Figure 5). PPI network analysis of DEGs was significantly enriched in GO terms. Line color represented the different types of interaction evidence.

**Figure 6:**
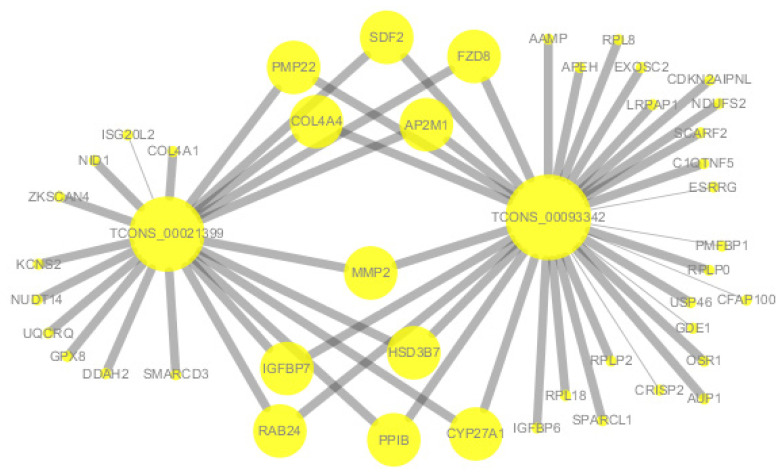
The lncRNA-mRNA co-expression network. The size of a node in the interaction network is proportional to the degree of the node. The thickness of inter-node lines is directly proportional to the value of Pearson’s correlation coefficient. Thicker lines indicate higher values.

According to the co-expression network, *TCONS-0002139* and *TCONS-00093342* were co-expressed with *MMP2* and collagen type IV alpha 4 chain (*COL4A4*). In addition, lncRNA *TCONS_00093342* was co-expressed with IGFBP6, and lncRNA *TCONS-0002139* was also co-expressed with *COL4A1* (Figure 6).

### Quantitative polymerase chain reaction validation

To test the reliability of our RNA-seq sequencing results, 11 differentially expressed mRNAs and five differentially expressed lncRNAs were used in validation through quantitative reverse-transcription PCR (qRT-PCR). The relative fold changes in the qRT-PCR assay showed that the qRT-PCR verification results were consistent with the RNA-seq sequencing results (Tables 2, 3).

### Western blotting validation

**Figure 7:**
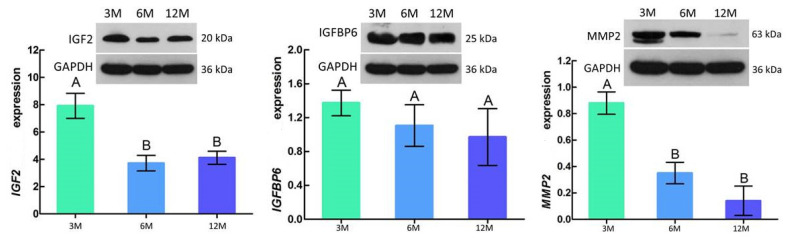
Relative abundance of IGF2, IGFBP6, and MMP2 protein in goat testes. Each sample was assessed three times. The results are represented by means±standard deviation. GAPDH was used as a loading control. 3M, 3 months old; 6M, 6 months old; 12M, 12 months old; IGF2, insulin-like growth factor 2; IGFBP6, insulin-like growth factor-binding protein 6; MMP2, matrix metalloproteinase 2; GAPDH, glyceraldehyde-3-phosphate dehydrogenase. ^A,B^ Different letters indicate significant differences (p<0.05); similar letters indicate that the difference is not significant (p>0.05).

Western blotting results showed that the protein expression trend of IGF2, IGFBP6, and MMP2 was consistent with the mRNA expression trend, with the highest expression levels at 3 months of age followed by a gradual decrease with increasing age. In addition, IGF2 and MMP2 were significantly different between 3 vs 6 months of age (p<0.05), with no significant difference between 6 vs 12 months of age (p>0.05) (Figure 7).

## DISCUSSION

lncRNAs, as new regulatory molecules in cell development, have attracted wide attention [28]. One reason is that lncRNAs may help regulate gene expression at transcriptional and post-transcriptional levels through genetic and epigenetic mechanisms [29]. In addition, they play a vital role in male fertility and spermatogenesis [30]. In recent years, reports employing lncRNA-seq technology have investigated the testicular tissue of ruminants at different developmental stages. For example, Yang et al [31] report a comparative analysis and study on lncRNAs and mRNAs during the maturation of sheep testis, which provides a valuable resource for further research regarding the functions of lncRNAs in ovine testicular development. Gao et al [32] conducted a comparative analysis of mRNAs and lncRNAs in prepubertal and post-pubertal bovine testicular tissues, which provides new information for further study of the biological functions of bovine lncRNAs.

There are also related studies regarding the expression of lncRNAs in different developmental stages of goat testis. Bo et al [33] employed RNA-seq in their study of the lncRNA of testicular tissues in prepubertal and pubertal goats. Their findings indicate that lncRNAs regulate different modes of spermatogenesis and testicular growth, and provide a new perspective for the analysis of lncRNA expression and age-related changes in goat testis [33]. However, the current study was limited to prepubertal and pubertal testicular tissue in goats, and lacked post-pubertal research. Therefore, we analyzed mRNA and lncRNA expression in the testicular tissues of goats aged 3, 6, and 12 months. We found the number of differentially expressed mRNAs was greater than that of differentially expressed lncRNAs. In addition, the number of DEGs between prepubertal and pubertal was higher than those between post-pubertal and pubertal. These findings were consistent with the findings in sheep testicles [34]. This phenomenon suggests that goat testicular tissue has a more complex gene regulation mechanism prior to puberty.

To further study the biological processes involved in testicular development and spermatogenesis, we functionally classified the differentially expressed mRNAs in the testicular tissues of goats at different developmental stages. The primary function of DEGs was elucidated by GO analysis. According to the GO term results, there was an evident and significant enrichment phenomenon between 3 vs 6 months of age. In addition, many of these GO terms were associated with the ECM, especially in terms of cellular components (Figure 3). Subsequently, PPI analysis was performed based on the significantly enriched GO terms at the age of 3 vs 6 months. Analysis results showed that these genes were mainly related to the ECM, including ECM structural constituent, ECM organization, and ECM-receptor interaction. In addition, according to PPI network, *MMP2* was the hub gene (Figure 4). *MMP2* was not only associated with the ECM, but also with the extracellular region (GO: 0005576). It is known that the ECM plays an essential role in forming the seminiferous tubule wall [35]. This phenomenon indicated that the age of 3 to 6 months was an essential period during formation of the extracellular region of goat testicular tissue, and that it is a critical stage in seminiferous tubule formation.

In the 3 vs 6 months GO terms, the number of DEGs in the extracellular region was the largest (Figure 3A). These genes included *MMP2*, *IGF2*, *IGFBP6*, and *COL4A1*. It is known that the testicular ECM is essential for the movement of germ cells through the blood-testis-barrier (BTB) during spermatogenesis; it is also known that proteins in the ECM modulate BTB dynamics through cytokines [36]. As a cytokine, IGF2 plays an essential regulatory role in the ECM [37]. In addition, many GO terms related to spermatogenesis were also enriched, including regulation of cell growth, IGF binding, cell growth, growth factor binding, and regulation of growth (Figure 3). These results further suggest that these genes regulate testicular development and spermatogenesis in goats through the ECM.

KEGG analysis of lncRNAs targets genes and DEGs revealed many pathways related to the ECM and spermatogenesis (Figure 5). These included focal adhesion, cell adhesion molecules, and ECM-receptor interaction. For example, the adhesion complex acts as an anchor junction between the germ cell epithelium and tissue ECM, regulating tight junctions between the Sertoli cells and germ cells as sperm cells pass through the germinal epithelium [36]. The association of lncRNAs with functionally annotated mRNAs can be achieved by co-expression networks [38]. We constructed a co-expression network of lncRNAs and their target genes to explore the potential regulatory mechanism of lncRNAs-mRNAs. The results of co-expression analysis showed that there were 11 mRNAs both co-expressing with lncRNA *Tcons_00021399* and *Tcons_0009334*, including *MMP2* (Figure 6). In addition, *COL4A1* was co-expressed with *Tcons_0021399*, and *IGFBP6* was co-expressed with *Tcons_0009334*. According to previous studies, these genes are particularly associated with the ECM in spermatogenesis; for example, *MMP2* is indispensable in the degradation and remodeling of the ECM [39] and *COL4A1* is closely associated with ECM recombination [40].

The remodeling process depends on the interaction between cells and the ECM [41]. *IGFBP6* is involved in the IGF regulatory pathway, which regulates the proliferation and differentiation of undifferentiated spermatogonia [42]. The IGF system is involved in various cellular biological functions, inducing the production of the ECM [43]. In addition, the expression of these genes is regulated by lncRNAs. For example, *LINC01128* plays a regulatory role in cell invasion, migration, and proliferation through the LINC01128/miR-299-3p/MMP2 axis [44]. Based on the above analysis, we speculated that the lncRNAs *Tcons_00021399* and *Tcons_0009334* may play an essential role in regulating molecular mechanisms related to ECM recombination or spermatogenesis in goat testis.

According to our results, the protein expression levels of IGF2, IGFBP6, and MMP2 were highest at 3 months of age, and then decreased with age (Table 3; Figure 8). IGF2 and MMP2 were significantly different between 3 vs 6 months of age (p<0.05). These results are similar to those found in the development of testicular tissue in mice [45]. In addition, IGF2 expression levels in the liver, kidney, and heart of male mice are significantly down-regulated with age [46], which was consistent with our findings in goat testicular tissues. These results further demonstrate the reliability of our transcriptome analysis from a protein perspective. However, the molecular mechanism between these lncRNAs and mRNAs needs further study.

## CONCLUSION

We studied mRNA and lncRNA expression levels using RNA-seq in the testicular tissues of goats at 3, 6, and 12 months of age. Several differentially expressed lncRNAs and mRNAs associated with the ECM and spermatogenesis were obtained. This study provides a high-quality resource for future goat transcriptome studies, especially for Guizhou Black goats. In addition, this study improves the comparative understanding of the molecular mechanisms of testicular development during goat sexual maturation.

## Figures and Tables

**Table 1:** List of the primers used in quantitative reverse-transcription polymerase chain reaction

NCBI Accession No	Gene	Primer sequence (5′–3′)	Tm	Product length
PRJNA613301	*TCONS_00020249*	TGAGTCCCTTGCGTCTCCTAT GTTGCCGAGACTGTGGTCA	60	270
PRJNA613301	*TCONS_00021399*	GCTCATGGTGGACTTGGTTC GCGTCTCGAATGGTGCTACT	60	184
PRJNA613301	*TCONS_00093342*	ATCCCAGATCCCGAGCGT CCAGTCAAAAGAAATACCACCCA	60	97
PRJNA613301	*TCONS_00124262*	CAGGGCACAGGCACAAGA TGTCCTGAGCATGTCCGAG	60	180
PRJNA613301	*TCONS_00070285*	TTGATGCCCTTCTTCTTCG TCACTGCGGTCCCTTCG	60	179
XM_005681882.3	*SPARCL1*	TTCTGAGCCTCTATTGGTGGA CTCGCTTGGGATGAAGTAGTC	58	127
XM_013973742.2	*CRISP2*	AAAACATACGAAAGGGCACAG AGAAACAGCACCAGTGGGAGT	60	102
XM_018040026.1	*HSD3B7*	TACACCGACATCCCTATCCTT GCCACGTTACCCACGTAGAC	59	244
XM_005693587.3	*PMP22*	CTTTATCACTCCCACATTTCCTTAT AGGTCTTGGAGTCTTGGCATC	58	194
XM_018056364.1	*COL4A1*	TCACAGGAGCTAAGGGAGATATG CTTGATGATGTTGAACGGACC	58	170
XM_018058852.1	*COL4A4*	CACAGTCAGACGGATGGAGAA AAGGGCAGCGTGCTAAAGA	59	155
XM_018066489.1	*HHIPL1*	GCATCAGCGAGTTCAGGGTC CGATTTGTTTTGGGCGTTTC	59	209
XM_018050766.1	*AMH*	TGACCGCAGACTCGGACTT GCTCAGGGGCTCACCATTT	60	113
XM_005691985.3	*MMP2*	TTGCTTCGTATGCACTTTGTTC GGAAACTGTTGAAGGGACTGG	59	268
NM_001314243.1	*IGFBP6*	GGGCGTACAAGACACTGAGATG CCCTATGGTCACAATTAGGCAC	59	122
NM_001287041.1	*IGF2*	TGCTGGTGCTTCTTGCCTTCT CGGCACAGTAGGTCTCCAGTAG	61	231
XM_005680968.3	*GAPDH*	TGGAGAAACCTGCCAAGTATGATGAG AGAGTGAGTGTCGCTGTTGAAGTCG	61	131

**Table 2:** List of lncRNA-seq and lncRNA-qPCR results

Item	Gene	3M	6M	12M
lncRNA-seq	*TCONS_00020249*	16.403±0.903^A^	10.934±0.959^B^	9.692±1.077^B^
	*TCONS_00021399*	13.982±1.176^A^	7.393±1.923^B^	5.979±1.298^B^
	*TCONS_00093342*	12.928±1.289^A^	6.098±1.183^B^	4.329±0.168^B^
	*TCONS_00124262*	5.290±0.886^A^	1.301±0.379^B^	2.401±0.893^AB^
	*TCONS_00070285*	11.145±1.282^A^	5.203±0.700^B^	5.595±1.067^B^
lncRNA-qPCR	*TCONS_00020249*	1.003±0.015^A^	0.596±0.002^B^	0.599±0.028^B^
	*TCONS_00021399*	1.001±0.003^A^	0.844±0.002^B^	0.492±0.002^C^
	*TCONS_00093342*	1.002±0.011^A^	0.300±0.048^B^	0.327±0.002^B^
	*TCONS_00124262*	0.998±0.007^A^	0.308±0.003^C^	0.501±0.004^B^
	*TCONS_00070285*	1.001±0.057^A^	0.667±0.002^B^	0.403±0.007^C^

qPCR, quantitative polymerase chain reaction; 3M, 3 months old; 6M, 6 months old; 12M, 12 months old.

A–C Different letters in the same row indicate significant differences (p<0.05).

**Table 3:** List of mRNA-seq and mRNA-qPCR results

Item	Gene	3M	6M	12M
mRNA-seq	*SPARCL1*	90.890±4.409^A^	52.901±3.092^B^	44.064±3.533^B^
	*CRISP2*	442.548±40.613^B^	646.077±30.079^A^	674.702±33.187^A^
	*HSD3B7*	7.596±0.656^A^	4.008±0.364^B^	3.592±0.390^B^
	*PMP22*	7.644±0.660^A^	3.664±0.602^B^	2.624±0.371^B^
	*COL4A1*	22.911±2.870^A^	10.757±1.118^B^	8.111±1.363^B^
	*COL4A4*	69.471±7.089^A^	27.079±1.636^B^	20.927±2.475^B^
	*HHIPL1*	6.534±0.361^A^	3.731±0.410^B^	2.822±0.382^B^
	*AMH*	8.897±0.975^A^	5.167±0.124^B^	4.944±0.488^B^
	*MMP2*	9.052±0.875^A^	4.427±0.422^B^	2.602±0.206^B^
	*IGFBP6*	130.894±9.796^A^	77.288±7.249^B^	69.930±9.074^B^
	*IGF2*	7.918±0.921^A^	3.719±0.571^B^	4.114±0.481^B^
mRNA-qPCR	*SPARCL1*	0.997±0.008^A^	0.376±0.011^B^	0.362±0.015^B^
	*CRISP2*	0.997±0.009^B^	1.270±0.009^A^	0.485±0.006^C^
	*HSD3B7*	1.002±0.009^A^	0.118±0.005^C^	0.148±0.004^B^
	*PMP22*	1.000±0.012^A^	0.491±0.026^B^	0.350±0.002^C^
	*COL4A1*	1.003±0.015^A^	0.031±0.001^B^	0.041±0.001^B^
	*COL4A4*	1.003±0.055^A^	0.596±0.024^B^	0.576±0.010^B^
	*HHIPL1*	1.003±0.010^A^	0.031±0.001^B^	0.046±0.002^B^
	*AMH*	1.002±0.008^A^	0.020±0.000^B^	0.011±0.000^B^
	*MMP2*	1.030±0.081^A^	0.520±0.317^A^	0.086±0.002^B^
	*IGFBP6*	1.001±0.047^A^	0.950±0.143^A^	0.486±0.017^A^
	*IGF2*	1.021 ±0.069^A^	0.519±0.024^B^	0.073±0.002^C^

qPCR, quantitative polymerase chain reaction; 3M, 3 months old; 6M, 6 months old; 12M, 12 months old.

A–C Different letters in the same row indicate significant differences (p<0.05).
